# Inference for Disattenuated Correlations

**DOI:** 10.1177/01466216261440511

**Published:** 2026-03-30

**Authors:** Jonas Moss

**Affiliations:** 1 6281 Department of Data Science and Analytics, BI Norwegian Business School, Oslo, Norway

**Keywords:** disattenuated correlation, measurement error, confidence intervals

## Abstract

When only summary statistics from published studies are available, the Hunter–Schmidt interval is the standard tool for inference on Spearman’s disattenuated correlation, but it treats reliability estimates as known constants and ignores their sampling variability. We derive a simple delta method variance that accounts for the uncertainty of all estimates while requiring nothing beyond the summaries already at hand. Under bivariate normality of scores and coefficient alpha from a normal parallel model, the corrected interval is asymptotically valid. In simulations it achieves coverage near nominal, while Hunter–Schmidt can undercover substantially when reliability is imprecisely estimated.

We are often interested in the correlation between two latent variables, $Z_{1}$ and $Z_{2}$ (e.g., conscientiousness and authoritarianism), which are not measured directly. Instead, they are estimated using scores, typically sum scores, derived from observed items. Denoting the scores by ${\hat{Z}}_{1}$ and ${\hat{Z}}_{2}$ and their reliabilities by $r_{1}$ and $r_{2}$ , Spearman’s formula (Spearman, 1904) gives the disattenuated correlation as

(1) $$\tau =Cor\left(Z_{1},Z_{2}\right)=\frac{Cor\left({\hat{Z}}_{1},{\hat{Z}}_{2}\right)}{\sqrt{r_{1}r_{2}}}=\frac{\rho }{\sqrt{r_{1}r_{2}}}.$$

Our focus is on inference for τ when only summary statistics from papers are available, namely, the sample correlation $\hat{\rho }$ , the estimated reliabilities ${\hat{r}}_{1}$ and ${\hat{r}}_{2}$ , their respective sample sizes $\left(n_{\rho },n_{1},n_{2}\right)$ , and potentially the number of items in each scale $\left(p_{1},p_{2}\right)$ .

In this setting, the most common approach is the Hunter–Schmidt confidence interval (Hunter & Schmidt, 2004):

(2) $$\frac{\hat{\rho }}{\sqrt{{\hat{r}}_{1}{\hat{r}}_{2}}}\pm \frac{1-{\hat{\rho }}^{2}}{\sqrt{n_{\rho }-1}\sqrt{{\hat{r}}_{1}{\hat{r}}_{2}}}z_{1-\alpha /2}.$$

However, the validity of this interval is questionable. Its variance estimator, $\frac{{\left(1-{\hat{\rho }}^{2}\right)}^{2}}{\left(n_{\rho }-1\right){\hat{r}}_{1}{\hat{r}}_{2}}$ , incorrectly treats the reliabilities ${\hat{r}}_{1}$ and ${\hat{r}}_{2}$ as known constants, thereby failing to account for their sampling error. This known limitation (Padilla & Veprinsky, 2012, 2014), along with the strict requirement of bivariate normal data for the variance of $\hat{\rho }$ (Fisher, 1915), motivates our goal of presenting a valid analytical confidence interval that incorporates the uncertainty of all estimates and is usable when only summary statistics are available. In practice, reliability estimates are often borrowed from an external validation study with its own, potentially small, sample size, making this source of uncertainty non-negligible, as illustrated in Example 1.

## The Method

To construct our corrected interval, we first derive a standard error for the disattenuated correlation that accounts for the sampling variability of all its components. Suppose one has independent estimates $\hat{\rho }$ , ${\hat{r}}_{1}$ and ${\hat{r}}_{2}$ for the correlation and reliabilities together with estimated standard errors $s_{\rho },s_{1}$ , and $s_{2}$ . Then a valid standard error for the disattenuated correlation is

(3) $$\sqrt{\hat{\Sigma }}=\sqrt{\frac{s_{\rho }^{2}}{{\hat{r}}_{1}{\hat{r}}_{2}}+\frac{1}{4}\frac{{\hat{\rho }}^{2}}{{\hat{r}}_{1}{\hat{r}}_{2}}\left(\frac{s_{1}^{2}}{{\hat{r}}_{1}^{2}}+\frac{s_{2}^{2}}{{\hat{r}}_{2}^{2}}\right)},$$

as may be found using a standard delta method argument. See the online appendix for a proof sketch.

While equation (3) provides a valid framework, its components ( $s_{\rho },s_{1}$ , and $s_{2}$ ) are not directly available from most published research. The formula applies to any reliability estimator with a known standard error, including omega or the greatest lower bound (e.g., McNeish, 2018; Sijtsma, 2009). To proceed from published summaries, however, we must adopt a model for the data that lets us calculate these standard errors from the information we do have. This requires three strong assumptions: (a) the scores are bivariate normal, (b) both reliabilities were estimated using coefficient alpha, and (c) the items follow a normal parallel model. Assumption (c) is needed not for alpha to consistently estimate reliability, as it does so under the weaker tau-equivalent model (Lord & Novick, 1968; Novick & Lewis, 1967), but for the standard error of alpha (Feldt, 1965; Kristof, 1963) to be correct. A more general standard error is available under the tau-equivalent model (van Zyl et al., 2000), but it requires the full item covariance matrix rather than the summary statistics we target here.

The utility of this approach is limited by the plausibility of these assumptions. Only assumption (b) typically holds, as coefficient alpha is still ubiquitous. However, the parallel model assumption is seldom met exactly, and psychometric data (often from Likert scales) are rarely truly normal. The substantial consequences of violating normality on the correlation are well-documented (e.g., Bishara et al., 2018; Kowalski, 1972). These effects are likely to be less pronounced here; however, since we are typically dealing with sum scores that are at least approximately marginally normal.

Under bivariate normality of scores we have $s_{\rho }=\left(1-{\hat{\rho }}^{2}\right)/\sqrt{n_{\rho }-1}$ , a result dating back to Fisher (1915). Under the parallel normal model we have $s_{1}=\sqrt{\frac{2p_{1}}{n_{1}\left(p_{1}-1\right)}}\left(1-{\hat{r}}_{1}\right)$ (Feldt, 1965; Kristof, 1963), where $p_{1}$ is the number of items, and likewise for $s_{2}$ . These may be plugged into equation (3) to obtain an estimated standard error. When $p_{1}$ and $p_{2}$ are known and all our assumptions are met we have

(4) $$\hat{\Sigma }=\frac{{\left(1-{\hat{\rho }}^{2}\right)}^{2}}{\left(n_{\rho }-1\right)\;{\hat{r}}_{1}{\hat{r}}_{2}}+\frac{{\hat{\rho }}^{2}}{2\;{\hat{r}}_{1}{\hat{r}}_{2}}\overset{2}{\underset{j=1}{\sum }}\frac{p_{j}}{n_{j}\left(p_{j}-1\right)}{\left(\frac{1-{\hat{r}}_{j}}{{\hat{r}}_{j}}\right)}^{2}.$$

Compared to the Hunter–Schmidt variance $\frac{{\left(1-{\hat{\rho }}^{2}\right)}^{2}}{\left(n_{\rho }-1\right){\hat{r}}_{1}{\hat{r}}_{2}}$ this formula contains an additional positive term.

Our corrected confidence interval is defined as

(5) $$\frac{\hat{\rho }}{\sqrt{{\hat{r}}_{1}{\hat{r}}_{2}}}\pm \sqrt{\hat{\Sigma }}\;z_{1-\alpha /2},$$

where $z_{1-\alpha /2}$ is the (1−α/2) -quantile of the standard normal distribution. The confidence intervals will be clipped to [−1,1] to respect the parameter space. R code (R Core Team, 2025) for these confidence intervals is provided as supplementary material. For the remainder of this note, all confidence intervals discussed below have level 95% .

From equation (3) we see that the confidence intervals can be widely different if one has small reliabilities, relatively high correlations, and small sample sizes for both the correlation and the reliabilities. For example, if $n_{\rho }=100$ , $n_{1}=100$ , $n_{2}=100$ , $r_{1}=r_{2}=0.5$ for p=5 , and $\hat{\rho }=0.6$ , then the Hunter–Schmidt confidence interval will be [0.9479,1] and the corrected [0.8357,1]. But the confidence intervals can also be quite similar, as in the following example.

Example 1 Neroni et al. (2022) reported a correlation of 0.38 between Self-Esteem (Rosenberg, 2016) and Perseverance of Effort (Duckworth et al., 2007) on a sample of $n_{\rho }=2544$ subjects. For self-esteem, Muslih and Chung (2024) reported $r_{1}=0.75$ , $n_{1}=260$ , and $p_{1}=4$ . For Perseverance of Effort, Christensen and Knezek (2014) reported $r_{2}=0.68$ , $n_{2}=152$ , and $p_{2}=6$ . Under the parallel model, the standard errors of these reliability estimates are approximately 0.025 and 0.040, respectively. The Hunter–Schmidt interval is [0.4855, 0.5787] and the corrected interval [0.4735, 0.5907].

Papers often report reliabilities and correlations from a single data set, which by necessity makes them correlated. Consequently, equation (4) is not strictly correct. However, in the online appendix we give a heuristic argument and simulation evidence suggesting that, under our stated assumptions, using equation (4) is conservative. This still supports using the corrected interval in practice.

Example 2 Marx and Winne (1978) reported correlations and alphas for three self-concept measures in one sample (n=488) of sixth-graders. For Gordon (p=5) versus Piers–Harris (p=5) the observed score correlation was $\hat{\rho }=0.57$ with alphas ${\hat{r}}_{1}=0.56$ and ${\hat{r}}_{2}=0.55$ . The Hunter–Schmidt interval is [0.9190,1] while the corrected interval is [0.8916,1]. Because the parameters are estimated on the same sample, the corrected interval is mildly conservative.

## Simulations

We first simulate under the normal parallel model, matching our plug-in assumptions, then examine robustness to the tau-equivalent model. We do not consider richer factor models. Under congeneric measurement, coefficient alpha can underestimate reliability, so alpha-based disattenuation can be biased. Fisher’s variance requires normality, and dropping it can make coverage arbitrarily poor for both intervals (see Duncan & Layard, 1973, equation (1.1)).

We ran 10,000 replicates with $p_{1}=p_{2}=5$ , τ∈{0.3,0.6,0.9} , reliability $r_{1}=r_{2}=a$ for a∈{0.60,0.90} , correlation sample size $n_{\rho }\in \left\{100,1000,5000\right\}$ , and reliability sample size $n_{1}=n_{2}=n_{a}\in \left\{100,1000,5000\right\}$ .

The results in Table 1 are simple. Hunter–Schmidt performs reasonably when $n_{a}$ is large relative to $n_{\rho }$ , but undercovers when reliability is noisy and $n_{\rho }$ is large. Higher τ exacerbates the problem. Where both intervals cover well, lengths are similar, so using the corrected interval by default sacrifices little.

**Table 1.** Confidence intervals using Hunter–Schmidt (HS) vs. Corrected (C)

			τ=0.30				τ=0.60				τ=0.90			
			HS		C		HS		C		HS		C	
α	$n_{\alpha }$	$n_{\rho }$	Cov	Len	Cov	Len	Cov	Len	Cov	Len	Cov	Len	Cov	Len
0.60	100	100	0.93	0.64	0.94	0.65	0.93	0.56	0.95	0.59	0.89	0.31	0.96	0.36
0.60	100	1000	0.93	0.20	0.96	0.23	0.82	0.18	0.96	0.27	0.64	0.12	0.96	0.23
0.60	100	5000	0.83	0.09	0.96	0.14	0.57	0.08	0.96	0.22	0.35	0.06	0.96	0.22
0.60	1000	100	0.94	0.63	0.94	0.63	0.94	0.56	0.94	0.56	0.93	0.33	0.94	0.33
0.60	1000	1000	0.94	0.20	0.95	0.20	0.94	0.18	0.95	0.19	0.91	0.14	0.95	0.16
0.60	1000	5000	0.94	0.09	0.95	0.09	0.89	0.08	0.95	0.10	0.77	0.07	0.95	0.11
0.60	5000	100	0.94	0.63	0.94	0.63	0.94	0.56	0.94	0.56	0.94	0.33	0.94	0.33
0.60	5000	1000	0.95	0.20	0.95	0.20	0.95	0.18	0.95	0.18	0.94	0.14	0.95	0.15
0.60	5000	5000	0.95	0.09	0.95	0.09	0.94	0.08	0.95	0.08	0.91	0.07	0.95	0.08
0.90	100	100	0.94	0.40	0.94	0.40	0.93	0.31	0.94	0.31	0.93	0.15	0.94	0.15
0.90	100	1000	0.95	0.13	0.95	0.13	0.94	0.10	0.95	0.10	0.83	0.05	0.95	0.07
0.90	100	5000	0.95	0.06	0.95	0.06	0.89	0.04	0.95	0.05	0.59	0.02	0.95	0.05
0.90	1000	100	0.94	0.40	0.94	0.40	0.94	0.31	0.94	0.31	0.94	0.15	0.94	0.15
0.90	1000	1000	0.95	0.13	0.95	0.13	0.95	0.10	0.95	0.10	0.93	0.05	0.94	0.05
0.90	1000	5000	0.95	0.06	0.95	0.06	0.94	0.04	0.94	0.04	0.90	0.02	0.95	0.03
0.90	5000	100	0.94	0.40	0.94	0.40	0.93	0.31	0.93	0.31	0.94	0.15	0.94	0.15
0.90	5000	1000	0.95	0.13	0.95	0.13	0.95	0.10	0.95	0.10	0.95	0.05	0.95	0.05
0.90	5000	5000	0.95	0.06	0.95	0.06	0.95	0.04	0.95	0.04	0.94	0.02	0.95	0.02

*Note.* Len = Confidence interval length, Cov = Coverage of confidence intervals. HS = Hunter–Schmidt interval, C = Corrected interval.

To assess sensitivity to violations of the parallel model assumption, we repeated the simulation under a tau-equivalent model (equal loadings, unequal error variances) with item variance ratios calibrated from the psych::bfi data (Revelle, 2026). Table 2 shows results for item variance ratios of approximately 1.9, representative of scales such as Openness in the BFI. A milder ratio of 1.3 gave nearly identical results (see the online appendix for details).

**Table 2.** Robustness under the tau-equivalent model (item variance ratio ≈1.9 )

			τ=0.30				τ=0.60				τ=0.90			
			HS		C		HS		C		HS		C	
α	$n_{\alpha }$	$n_{\rho }$	Cov	Len	Cov	Len	Cov	Len	Cov	Len	Cov	Len	Cov	Len
0.60	100	100	0.94	0.65	0.94	0.66	0.92	0.56	0.95	0.59	0.89	0.31	0.96	0.36
0.60	100	1000	0.92	0.20	0.96	0.23	0.83	0.18	0.96	0.27	0.64	0.12	0.96	0.23
0.60	100	5000	0.83	0.09	0.97	0.14	0.57	0.08	0.96	0.22	0.35	0.06	0.96	0.22
0.60	1000	100	0.94	0.63	0.94	0.63	0.94	0.56	0.95	0.56	0.93	0.33	0.94	0.33
0.60	1000	1000	0.95	0.20	0.95	0.20	0.94	0.18	0.95	0.19	0.91	0.14	0.95	0.16
0.60	1000	5000	0.94	0.09	0.95	0.09	0.90	0.08	0.95	0.10	0.77	0.07	0.95	0.11
0.60	5000	100	0.94	0.63	0.94	0.63	0.94	0.56	0.94	0.56	0.94	0.33	0.94	0.33
0.60	5000	1000	0.95	0.20	0.95	0.20	0.95	0.18	0.95	0.18	0.94	0.14	0.95	0.15
0.60	5000	5000	0.94	0.09	0.95	0.09	0.94	0.08	0.95	0.08	0.92	0.07	0.95	0.08
0.90	100	100	0.94	0.40	0.94	0.40	0.94	0.31	0.94	0.31	0.92	0.15	0.94	0.15
0.90	100	1000	0.95	0.13	0.95	0.13	0.94	0.10	0.95	0.10	0.84	0.05	0.95	0.07
0.90	100	5000	0.94	0.06	0.95	0.06	0.89	0.04	0.95	0.05	0.60	0.02	0.95	0.05
0.90	1000	100	0.94	0.40	0.94	0.40	0.94	0.31	0.94	0.31	0.94	0.15	0.94	0.15
0.90	1000	1000	0.95	0.13	0.95	0.13	0.95	0.10	0.95	0.10	0.94	0.05	0.95	0.05
0.90	1000	5000	0.94	0.06	0.94	0.06	0.95	0.04	0.95	0.05	0.89	0.02	0.95	0.03
0.90	5000	100	0.94	0.40	0.94	0.40	0.94	0.31	0.94	0.31	0.94	0.15	0.94	0.15
0.90	5000	1000	0.95	0.13	0.95	0.13	0.95	0.10	0.95	0.10	0.94	0.05	0.95	0.05
0.90	5000	5000	0.95	0.06	0.95	0.06	0.95	0.04	0.95	0.04	0.94	0.02	0.95	0.02

*Note.* Len = Confidence interval length, Cov = Coverage. HS = Hunter–Schmidt, C = Corrected.

In this tau-equivalent setting, the corrected interval maintains coverage near 95% , while Hunter–Schmidt undercovers when $n_{\rho }$ is large relative to $n_{a}$ .

To probe the normality assumption, we also ran a reduced-design simulation with standardized t(5) latent factors and item errors (full results in the online appendix). Non-normality reduces coverage for both intervals, with neither reaching nominal at high τ . At τ=0.90 and α=0.60 , for example, corrected coverage ranged from 0.87 to 0.92 across design conditions while Hunter–Schmidt ranged from 0.52 to 0.82.

## Concluding Remarks

We propose a corrected confidence interval for disattenuated correlations that accounts for the sampling variability of the reliability estimates. It is necessarily wider than the Hunter–Schmidt interval, but its empirical coverage is close to nominal across conditions. When only summary statistics are available (e.g., in meta-analyses), the corrected interval is a sensible default. But with raw data, latent-variable models such as lavaan (Rosseel, 2012) are preferable, and bootstrap intervals for the disattenuated correlation have also performed well in prior work (Padilla & Veprinsky, 2012, 2014).

Our approach assumes bivariate normality of sum scores and a normal parallel model. Bivariate normality may be violated, which can reduce coverage (Kowalski, 1972). Under non-parallel measurement, the coefficient alpha standard error of Kristof (1963) and Feldt (1965) is typically underestimated, again hurting coverage. Although equation (3) admits alternative standard errors, robust choices require raw data and are biased in small samples (Xiao & Hau, 2022). In our summary statistics setting, these assumptions cannot be directly verified, but researchers can consult the source studies for item-level diagnostics. In practice, researchers should look for roughly symmetric sum-score distributions, no strong piling up at the lowest or highest response categories, and item variances that are not wildly different. When such diagnostics are unavailable, the interval should be viewed as the best available large-sample correction to Hunter–Schmidt under limited information. As shown in the simulation study, the corrected interval is reasonably robust to realistic tau-equivalent departures from parallelism, but non-normality can reduce coverage for both intervals. The corrected interval is not assumption-free, but it is the best that can be done without raw data.

## Supplemental Material

**Supplemental Material -** Inference for Disattenuated Correlations Supplemental Material for Inference for Disattenuated Correlations by Jonas Moss in Applied Psychological Measurement.

## Data Availability

Provided in the supplemental material.*
