# Supplementary material for: Inference for Disattenuated Correlations
Source: Appl Psychol Meas. 2026 Mar 30:01466216261440511. Online ahead of print. doi: 10.1177/01466216261440511 (PMC13035683; doi:10.1177/01466216261440511)
Supplement: Supplemental Material - Inference for Disattenuated Correlations [file sj-pdf-1-apm-10.1177_01466216261440511.pdf]

Online appendix for "Inference for disattenuated correlations"

March 13, 2026

1 Proof sketches

The validity of our proposed standard error can be proved rigorously using standard asymptotic theory (van der Vaart 2000), using the delta rule and Slutsky's theorem while accounting for different sample sizes.

Recall the standard deviation

$$\sqrt{\hat{\Sigma}} = \sqrt{\frac{s_\rho^2}{\hat{r}_1\hat{r}_2} + \frac{1}{4}\frac{\hat{\rho}^2}{\hat{r}_1\hat{r}_2}\left(\frac{s_1^2}{\hat{r}_1^2} + \frac{s_2^2}{\hat{r}_2^2}\right)}. \tag{1}$$

Our function is the disattenuated correlation, which depends on three estimated quantities we treat as independent random variables: $\hat{\tau} = f(\hat{\rho}, \hat{r}_1, \hat{r}_2)$, where $f(\rho, r_1, r_2) = \frac{\rho}{\sqrt{r_1 r_2}}$. For a function of multiple independent variables, the variance of the function is approximately the sum of the variances contributed by each variable. The contribution of each variable is its own variance multiplied by the square of the partial derivative of the function with respect to that variable, evaluated at the sample estimates.

$$\text{Var}(\hat{\tau}) \approx \left(\frac{\partial\tau}{\partial\rho}\right)^2 \text{Var}(\hat{\rho}) + \left(\frac{\partial\tau}{\partial r_1}\right)^2 \text{Var}(\hat{r}_1) + \left(\frac{\partial\tau}{\partial r_2}\right)^2 \text{Var}(\hat{r}_2).$$

The partial derivatives are

$$\begin{aligned}
\frac{\partial\tau}{\partial\rho} &= \frac{1}{\sqrt{r_1 r_2}}, \\
\frac{\partial\tau}{\partial r_1} &= -\frac{1}{2r_1}\frac{\rho}{\sqrt{r_1 r_2}} = -\frac{\tau}{2r_1}, \\
\frac{\partial\tau}{\partial r_2} &= -\frac{1}{2r_2}\frac{\rho}{\sqrt{r_1 r_2}} = -\frac{\tau}{2r_2}.
\end{aligned}$$

We substitute these derivatives back into the approximation formula, using the sample estimates for each parameter $(\hat{\rho}, \hat{r}_1, \hat{r}_2)$ and their estimated variances (s_ρ^2, s_1^2, s_2^2):

$$\mathrm{Var}(\hat{\tau}) \approx \frac{1}{\hat{r}_1\hat{r}_2} s_\rho^2 + \left(-\frac{\hat{\tau}}{2\hat{r}_1}\right)^2 s_1^2 + \left(-\frac{\hat{\tau}}{2\hat{r}_2}\right)^2 s_2^2.$$

Simplifying this gives the estimated variance

$$\hat{\Sigma} = \frac{s_\rho^2}{\hat{r}_1\hat{r}_2} + \frac{\hat{\tau}^2}{4}\left(\frac{s_1^2}{\hat{r}_1^2} + \frac{s_2^2}{\hat{r}_2^2}\right).$$

The standard error for the disattenuated correlation is the square root of this variance, $\sqrt{\hat{\Sigma}}$. This provides the justification for the standard deviation formula.

The specialized formula

$$\hat{\Sigma} = \frac{(1-\hat{\rho}^2)^2}{(n_\rho - 1)\hat{r}_1\hat{r}_2} + \frac{1}{2}\frac{\hat{\rho}^2}{\hat{r}_1\hat{r}_2}\left(n_1^{-1}\frac{p_1}{p_1 - 1}\left(\frac{1-\hat{r}_1}{\hat{r}_1}\right)^2 + n_2^{-1}\frac{p_2}{p_2 - 1}\left(\frac{1-\hat{r}_2}{\hat{r}_2}\right)^2\right). \tag{2}$$

follows from plugging in the stated definitions of the standard errors under the normal parallel model.

2 Conservatism

When $\hat{\rho}$, $\hat{r}_1$, and $\hat{r}_2$ are all calculated from the same sample they are not independent. The full variance approximation from the delta method must include covariance terms:

$$\begin{aligned}\mathrm{Var}(\hat{\tau}) \approx V_{\text{ind}} &+ 2\frac{\partial\tau}{\partial\rho}\frac{\partial\tau}{\partial r_1}\mathrm{Cov}(\hat{\rho}, \hat{r}_1), \\ &+ 2\frac{\partial\tau}{\partial\rho}\frac{\partial\tau}{\partial r_2}\mathrm{Cov}(\hat{\rho}, \hat{r}_2), \\ &+ 2\frac{\partial\tau}{\partial r_1}\frac{\partial\tau}{\partial r_2}\mathrm{Cov}(\hat{r}_1, \hat{r}_2),\end{aligned}$$

where V_{ind} is the variance assuming independence. Our proposed method uses V_{ind} and ignores the three additional covariance terms. The discussion below is intended as a heuristic for why this may still lead to a conservative interval, that is, an interval with coverage at or above the nominal level. The idea is that (i) the sum of the first two covariance terms is plausibly negative and (ii) the final covariance term may be small when n is large.

Now we examine the signs of these terms, assuming the true latent correlation $\tau > 0$ for simplicity, as the logic is identical if $\tau < 0$. Consider first the sign of $\mathrm{Cov}(\hat{\rho}, \hat{r}_k)$, which we claim is positive. Intuitively, if sampling error leads to a sample where items are more consistent than they are in the population, this will inflate our estimate of reliability $\hat{r}_k$. This same increase in item consistency will also tend to inflate the correlation of that scale's total score with other variables, thereby inflating $\hat{\rho}$. Thus, we expect $\mathrm{Cov}(\hat{\rho}, \hat{r}_k) > 0$.

From our main derivation, we know that (a) $\partial\tau/\partial\rho = 1/\sqrt{r_1 r_2} > 0$, and (b) $\partial\tau/\partial r_k = -\tau/(2r_k) < 0$, since we assumed $\tau > 0$. The sign of the first two covariance terms in the variance expansion is therefore the product of the signs of its components, which is negative.

We consider $\mathrm{Cov}(\hat{r}_1, \hat{r}_2)$ next. The reliability $\hat{r}_1$ is calculated using only the items for scale 1, while $\hat{r}_2$ uses only the items for scale 2. Because these two sets of items are distinct, the random sampling errors affecting their respective covariance matrices are asymptotically independent. Therefore, the covariance between the two reliability estimates, $\mathrm{Cov}(\hat{r}_1, \hat{r}_2)$, is small and approaches zero as the sample size grows.

In conclusion, the full variance is heuristically

$$\mathrm{Var}(\hat{\tau}) \approx V_{\mathrm{ind}} - (\text{a positive quantity}) + (\text{a small quantity}).$$

This suggests that using V_{ind} may produce a confidence interval that is somewhat too wide, and hence conservative, under the normal parallel model.

We examined this heuristic by simulation under the normal parallel model. We generated both scales and the correlation from a single sample of size n, computed $\hat{\rho}$, $\hat{r}_1$, and $\hat{r}_2$ from the same data, and then applied the corrected interval as if they were independent. Table 1 reports coverage and average length over 10,000 replicates with $p_1 = p_2 = 5$. The corrected interval is near nominal and often conservative in these conditions, which is consistent with conservatism of the independence-based variance under the normal parallel model.

3 Tau-equivalent simulation details

The tau-equivalent simulation in Table 2 of the main text uses item variance ratios calibrated from the `psych::bfi` data (Revelle 2026), which contains five-item scales for the Big Five personality traits. Item variance ratios (max/min) across scales range from approximately 1.1 (Neuroticism) to 1.9 (Openness). We chose two representative configurations with $p = 5$ items and error variances proportional to:

Table 1: Coverage and length of the corrected interval when all estimates come from one sample ($p_1 = p_2 = 5$).

α	n	τ	Cov	Len
0.60	100	0.3	0.95	0.65
0.60	100	0.6	0.97	0.59
0.60	100	0.9	0.99	0.37
0.60	500	0.3	0.95	0.29
0.60	500	0.6	0.97	0.27
0.60	500	0.9	0.99	0.21
0.60	1000	0.3	0.95	0.20
0.60	1000	0.6	0.97	0.19
0.60	1000	0.9	0.99	0.16
0.90	100	0.3	0.94	0.40
0.90	100	0.6	0.95	0.31
0.90	100	0.9	0.97	0.16
0.90	500	0.3	0.95	0.18
0.90	500	0.6	0.96	0.14
0.90	500	0.9	0.98	0.07
0.90	1000	0.3	0.95	0.13
0.90	1000	0.6	0.96	0.10
0.90	1000	0.9	0.98	0.05

Note. Cov = coverage, Len = average interval length. All estimates ($\hat{\rho}$, $\hat{r}_1$, $\hat{r}_2$) computed from the same sample of size n.

- *Mild* (≈ 1.3): $(0.87, 0.93, 1.00, 1.07, 1.13)$, representative of Neuroticism.
- *Moderate* (≈ 1.9): $(0.70, 0.85, 1.00, 1.15, 1.30)$, representative of Openness.

All items share a common loading λ chosen so that the population reliability equals the target α. Table 2 in the main text reports the moderate condition. Table 2 below reports the mild condition. Results are nearly identical.

4 Heavy-tailed non-normality

Because Fisher's variance for the sample correlation is exact only under normality, departures from normality can affect both intervals (Kowalski 1972). As a minimal robustness check, we repeated the parallel-model simulation af-

Table 2: Tau-equivalent simulation, mild item variance ratio (≈ 1.3).

			$\tau = 0.30$				$\tau = 0.60$				$\tau = 0.90$			
			HS		C		HS		C		HS		C	
α	n_α	n_ρ	Cov	Len	Cov	Len	Cov	Len	Cov	Len	Cov	Len	Cov	Len
0.60	100	100	0.93	0.64	0.94	0.65	0.93	0.56	0.95	0.58	0.89	0.31	0.96	0.36
0.60	100	1000	0.92	0.20	0.95	0.23	0.82	0.18	0.97	0.27	0.65	0.12	0.96	0.23
0.60	100	5000	0.83	0.09	0.96	0.14	0.56	0.08	0.97	0.22	0.34	0.06	0.96	0.22
0.60	1000	100	0.94	0.63	0.94	0.63	0.94	0.56	0.94	0.56	0.93	0.33	0.94	0.33
0.60	1000	1000	0.95	0.20	0.95	0.20	0.94	0.18	0.95	0.19	0.91	0.14	0.95	0.16
0.60	1000	5000	0.94	0.09	0.95	0.09	0.89	0.08	0.95	0.10	0.78	0.07	0.95	0.11
0.60	5000	100	0.94	0.63	0.94	0.63	0.94	0.56	0.94	0.56	0.94	0.33	0.94	0.33
0.60	5000	1000	0.95	0.20	0.95	0.20	0.95	0.18	0.95	0.18	0.94	0.14	0.95	0.15
0.60	5000	5000	0.95	0.09	0.95	0.09	0.94	0.08	0.95	0.08	0.91	0.07	0.95	0.08
0.90	100	100	0.94	0.40	0.94	0.40	0.94	0.31	0.94	0.31	0.92	0.15	0.94	0.15
0.90	100	1000	0.94	0.13	0.95	0.13	0.94	0.10	0.95	0.10	0.84	0.05	0.95	0.07
0.90	100	5000	0.94	0.06	0.95	0.06	0.89	0.04	0.95	0.05	0.60	0.02	0.95	0.05
0.90	1000	100	0.94	0.40	0.94	0.40	0.94	0.31	0.94	0.31	0.94	0.15	0.94	0.15
0.90	1000	1000	0.95	0.13	0.95	0.13	0.95	0.10	0.95	0.10	0.94	0.05	0.95	0.05
0.90	1000	5000	0.95	0.06	0.94	0.06	0.94	0.04	0.95	0.05	0.90	0.02	0.95	0.03
0.90	5000	100	0.94	0.40	0.94	0.40	0.94	0.31	0.94	0.31	0.94	0.15	0.94	0.15
0.90	5000	1000	0.95	0.13	0.95	0.13	0.95	0.10	0.95	0.10	0.95	0.05	0.95	0.05
0.90	5000	5000	0.95	0.06	0.95	0.06	0.95	0.04	0.95	0.04	0.94	0.02	0.95	0.02

Note. Cov = coverage, Len = average interval length. HS = Hunter–Schmidt, C = Corrected.

ter replacing all Gaussian latent factors and item errors with standardized $t(5)$ variables. This preserves the parallel measurement structure and the independent reliability samples while introducing heavy tails. The design is intentionally reduced to $(n_\rho, n_\alpha) \in \{(100, 100), (1000, 100), (1000, 1000)\}$ with $p = 5$. This is not intended as a realistic model for all psychometric non-normality, only as a simple stress test.

Table 3 shows that heavy tails reduce coverage for both intervals, especially when τ is large. Even so, the corrected interval remains materially closer to nominal than Hunter–Schmidt in the settings where reliability uncertainty matters most, namely when n_ρ is large relative to n_α. Under non-normality the corrected interval is therefore not fully robust, but it still improves substantially on Hunter–Schmidt in the cases that motivate the paper.

Table 3: Heavy-tailed robustness under standardized $t(5)$ factors and item errors.

α	n_α	n_ρ	τ	HS Cov	C Cov	HS Len	C Len
0.60	100	100	0.30	0.93	0.94	0.65	0.66
0.60	100	100	0.60	0.89	0.92	0.55	0.58
0.60	100	100	0.90	0.82	0.91	0.31	0.36
0.60	100	1000	0.30	0.89	0.94	0.21	0.24
0.60	100	1000	0.60	0.74	0.93	0.19	0.28
0.60	100	1000	0.90	0.52	0.92	0.11	0.23
0.60	1000	1000	0.30	0.93	0.94	0.20	0.20
0.60	1000	1000	0.60	0.89	0.90	0.18	0.19
0.60	1000	1000	0.90	0.81	0.87	0.14	0.15
0.90	100	100	0.30	0.92	0.92	0.40	0.40
0.90	100	100	0.60	0.88	0.88	0.31	0.31
0.90	100	100	0.90	0.82	0.85	0.15	0.15
0.90	100	1000	0.30	0.93	0.93	0.13	0.13
0.90	100	1000	0.60	0.85	0.87	0.10	0.10
0.90	100	1000	0.90	0.66	0.82	0.05	0.07
0.90	1000	1000	0.30	0.93	0.93	0.13	0.13
0.90	1000	1000	0.60	0.87	0.87	0.10	0.10
0.90	1000	1000	0.90	0.80	0.82	0.05	0.05

Note. Cov = coverage, Len = average interval length. HS = Hunter–Schmidt, C = Corrected.

References

Kowalski, C. J. (1972). On the effects of non-normality on the distribution of the sample product-moment correlation coefficient. *Journal of the Royal Statistical Society. Series C, Applied Statistics*, *21*(1), 1. https://doi.org/10.2307/2346598

Revelle, W. (2026). *Psych: Procedures for psychological, psychometric, and personality research* [R package version 2.6.1]. Northwestern University. Evanston, Illinois. https://CRAN.R-project.org/package=psych

van der Vaart, A. W. (2000). *Asymptotic statistics*. Cambridge University Press.
